# The mortality burden of hematological malignancies in Ecuador

**DOI:** 10.3126/nje.v11i2.37057

**Published:** 2021-06-30

**Authors:** David Garrido, Andrés Orquera, Johanna Rojas, Manuel Granja

**Affiliations:** 1 Posgrado de Hematología, Hospital de Clínicas “Dr. Manuel Quintela”, Universidad de la República, Av Italia, Montevideo, Uruguay; 2 Hematology Department, Hospital Carlos Andrade Marín, Av. Universitaria, Quito, Ecuador; 3 Hematology Department, Hospital Metropolitano, Av. Mariana de Jesús s/n, 170521, Quito, Ecuador; 4 Hematology Department, Hospital Carlos Andrade Marín, Av. Universitaria, Quito, Ecuador

**Keywords:** Hematological neoplasm, Lymphoma, Leukemia, Observational studies, Latin America

## Abstract

**Background:**

The Hematological neoplasms (HN) are a heterogeneous group of cancers that originated in the hematopoietic or lymphoid tissues. There is reduced information published regarding HN mortality in Ecuador. This study aims to present the crude and age-specific mortality rates for HN in the Ecuadorian population.

**Methods:**

We performed a cross-sectional study through the national database of defunctions published by the Ecuadorian National Institute of Statistics and Census, 2019. We used the ICD-10 codes to classify the HN.

**Results:**

During 2019, 1462 deaths were reported, 53.83% were males, 87.96% of mestizo ethnicity, and 78.32% residents in urban areas. The median age was 62 years, with an interquartile range of 34.

The crude mortality rate obtained was 8.49 per 100000 inhabitants, and the higher age-specific mortality rates was 43.29 per 100000 inhabitants aged ≥ 60 years, contrasting with the 2.63 per 100000 inhabitants in people aged < 20 years. Considering each ICD-10 group, we found the following rates by 100000 inhabitants; C85 2.04, C91 1.92, C92 1.46, C90 1.11, C83 0.70, C95 0.48, C81 0.38, C84 0.16, C82 0.10, C96 0.05, C93 0.04, C86 and C94 0.02, and C88 0.01.

**Conclusion:**

In Ecuador, during 2019, approximately eight people died due to HN by 100000 inhabitants, affecting mainly people aged ≥ 60 years. The most frequent neoplasms were Non-Hodgkin lymphomas, similar to other reports globally. These results should be analyzed considering some deficiencies in the Ecuadorian health system and the national registry. Therefore, we suggest conducting more studies regarding HN.

## Introduction

The term “Hematological malignancies” (HM) defines a diverse group of neoplastic diseases originated in the hematopoietic and lymphoid tissues, with a particular cytogenetic profile and clinical presentation depending each case and cell lineage [1].

In 2016 the World Health Organization published an updated version of the classification of tumors of the hematopoietic and lymphoid tissues, including more than 100 clinical entities [2].

HN represents 6.5% of all cancers globally, and particularly in Latin America, it has been observed that 57.7% of HN are represented by Non-Hodgkin Lymphoma (NHL), 29.5% Multiple Myeloma (MM), and 12.7% Chronic Lymphocytic Leukemia (CLL) [3, 4].

There is limited information regarding the mortality by HN in the Ecuadorian population. Therefore, this study aims to present the mortality analysis for these pathologies through the national registry of deaths corresponding to 2019.

## Methodology

We performed an observational and cross-sectional study, reporting the crude and age-specific death rates by HN in the Ecuadorian population in 2019.

### Location

**Figure 1.**
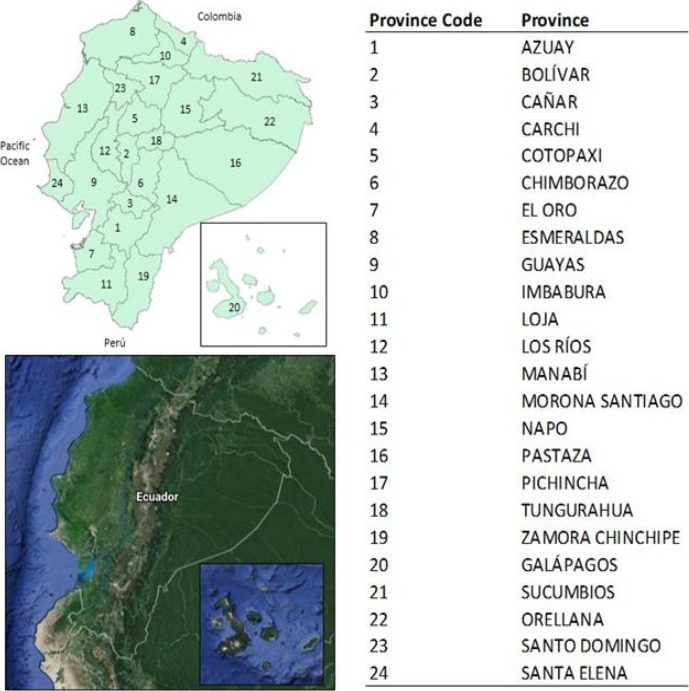
Administrative Map of Ecuador

Ecuador is a country located in the northwest of South America and borders Colombia to the North, the Pacific Ocean to the West, and Peru to the South and East. The country crosses the Equatorial line and has approximately 17 million inhabitants living in 283 560 km2. It is administratively divided into 24 provinces, distributed in four regions, Andean (Sierra), Coastal (Costa), Amazon (Oriente), and Insular (Galapagos) (Figure 1).

### Data collection

Data were obtained from the governmental database published in 2019 [last year published] by the Ecuadorian National Institute of Statistics and Census (INEC), the last published by the institution. The information is presented by INEC in SPSS format and is freely available on their website.

The datasets analyzed during the current study are entitled “Anuario de Nacimientos y Defunciones” (https://www.ecuadorencifras.gob.ec/anuario-de-nacimientos-y-defunciones/).

As the SPSS report has data on all the deaths reported in the country during 2019, we used the ICD-10 codification to find the patients affected by hematological neoplasms.

The ICD-10 codes, which correspond to hematological neoplasms, range from C81 to C96.

For this study, we included the following information: the total number of deaths reported by hematological neoplasms each year, the total number of inhabitants each year by province, and demographic characteristics, including age, sex, and province of residence.

### Calculation

We performed the statistical analysis using the software SPSS V.25 and Excel 2013. Qualitative variables were presented as percentages and quantitative variables as median values with interquartile interval.

To calculate the crude mortality rate, we used the equation:

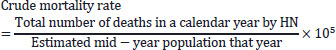


To calculate the age adjusted mortality rate, we used the equation:

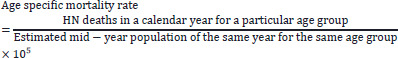


Years of life lost (YLL) due to premature mortality (YLLs) were calculated using the following equation: [5]

YLL=Number of deaths by a specific HN ×Life expectancy at the age of death

Because the database provided by INEC does not present the date of diagnosis, we cannot include the calculation of disability-adjusted life years (DALY) and years lived with disability (YLDs). As described by INEC in the document “MUJERES Y HOMBRES del Ecuador en Cifras III”, we considered life expectancy of 74.3 years for the male population and 80.2 for females.

To compare the mortality rates between regions, we used the mean differences (MD), which was presented along with confidence intervals (CI) and significance level through p-value.

## Results

According to the data published by the National Institute of Statistics and Censuses (INEC), during 2019, 1462 deaths were associated with hematological neoplasms. From them, 787 (53.83%) were males, 1286 (87.96%) consider themselves to be of mestizo ethnicity, 1145 (78.32%) lived in the urban area. Additionally, the median age was 62 years, with an interquartile range (IQR) of 34. For male patients, the median age was 63 years (IQR 35), and for females, the median age was 62 years (IQR 33).

**Figure 2.**
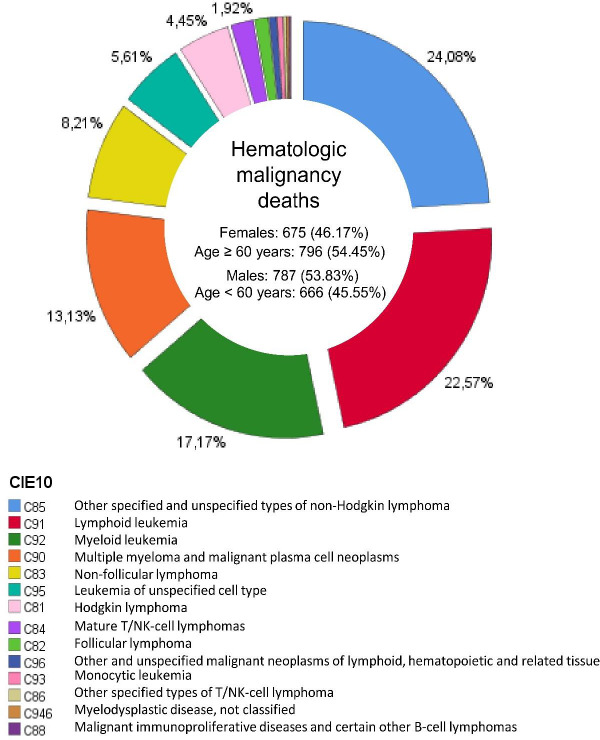
Percentage of patients deceased classified by ICD-10 code

With regards to the HN diagnosed, in order of frequency, the pathologies included were diagnosed as follows; 352 (24.08%) ICD-10 C85 [Other and unspecified types of non-Hodgkin lymphoma], 330 (22.57%) ICD-10 C91 [Lymphoid leukemia], 251 (17.17%) ICD-10 C92 [Myeloid leukemia], 192 (13.13%) ICD-10 C90 [Multiple myeloma and malignant plasma cell neoplasms], and 120 (8.21%) ICD-10 C83 [Non-Follicular lymphoma] (Figure 2). The detailed results are presented in Table 1.

Regarding age in the different neoplasms, the highest age was found in patients with ICD-10 C93 [Monocytic leukemia] with 79 years, and the lower median age corresponds to patients with ICD-10 C91, with 37.5 years. However, most patients in this group (277/330) were diagnosed with ICD-10 C91.0 [Acute lymphoblastic leukemia].

Male patients were affected more frequently in the majority of diseases, excepting ICD-10 C83.5 [Lymphoblastic lymphoma], ICD-10 C83.9 [Other non-follicular lymphoma], ICD-10 C90.1 [Plasma cell leukemia], ICD-10 C90.3 [Solitary plasmacytoma], ICD-10 C91.0 [Acute lymphoblastic leukemia], ICD-10 C92.3 [Myeloid sarcoma], ICD-10 C92.4 [Acute promyelocytic leukemia], and ICD-10 C92.5 [Acute myelomonocytic leukemia].

### Mortality rates

The crude mortality rate obtained was 8.49 per 100000 inhabitants, and the age-specific mortality rates were 43.29 per 100000 inhabitants aged ≥ 60 years, 9.32 per 100000 inhabitants aged 40 to 59 years, 3.15 per 100000 inhabitants aged 20 to 39 years, and 2.63 per 100000 inhabitants aged < 20 years (Table 2). The mortality rate was significantly higher for the population with 60 years of age or more (p<0.05).

Considering each ICD-10 group, we found the following rates by 100000 inhabitants; C85 2.04, C91 1.92, C92 1.46, C90 1.11, C83 0.70, C95 0.48, C81 0.38, C84 0.16, C82 0.10, C96 0.05, C93 0.04, C86 and C94 0.02, and C88 0.01.

### Comparison between regions

**Figure 3.**
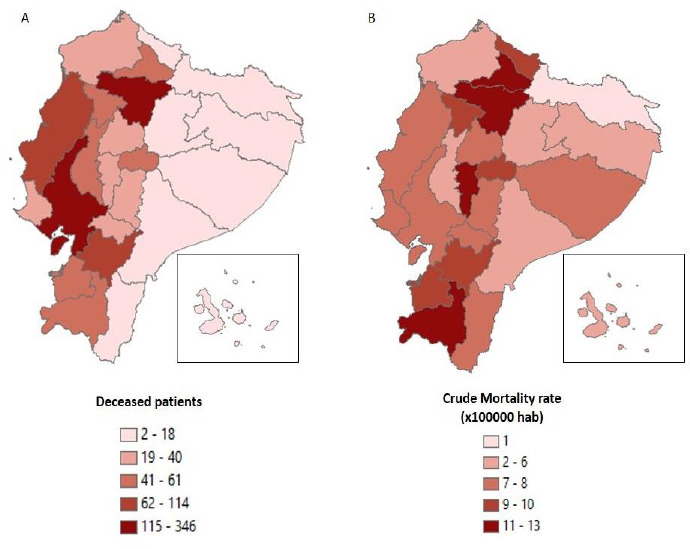
Number of deceased patients (A), and crude mortality rates by province of residence (B)

The highest average mortality rate corresponds to Andean Region with 9.81 deaths by 100000 inhabitants, followed by Coastal Region (7.70/100000) and Amazon Region (4.78/100000). Mortality rates by province of residence are presented in Figure 3.

The MD was significant for all comparisons, including; Andean Region vs. Coastal Region (MD -2.11, 95% CI -3.78 to -0.42, p<0.05), Andean Region vs. Amazon Region (MD -5.03, CI -7.07 to -2.99, p<0.05), and Coastal Region vs. Amazon Region (MD -2.92, CI -5.14 to -0.70, p<0.05).

## Discussion

As estimated by GLOBOCAN 2018, there were 18.1 million new cases and 9.6 million cancer deaths worldwide, from which 248,724 (2.6%), 309,006 (3.2%), 106,105 (1.1%), and 26,167 (0.3%) deaths corresponds to NHL, Leukemia, MM, and Hodgkin lymphoma (HL), respectively [6].

In our study, we found that the crude mortality rate for HN was 8.49 per 100000 inhabitants, with the highest number of deceased patients corresponding to NHL, leukemia, and MM. This results are similar whit other reports presented in the region.

In Central and South America, regarding NHL, the reported age-standardized mortality rates per 100,000 varied from 1.3 to 9.2 in female patients and 1.4 to 10.9 among males [7].

In an analysis conducted through data corresponding to 17 countries from the World Health Organization, for the period 1995–2013, mortality rates were higher in male population, the highest MM mortality rates were registered in Chile (15.1/100,000 in men and 11.9/100,000 in women), and the majority of countries presented increasing trends with the highest increments in Guatemala, Ecuador, Paraguay, and Brazil [8].

In Latin America from 1990 to 2017, for AML, it has been reported that El Salvador and Ecuador had the most rapid increase in Age-Standardized Death Rate (ASDR), with estimated annual percentage changes of 3.62 (95% CI 2.93 to 4.31) and 3.53 (95% CI 3.13 to 3.93), respectively [9]. Similarly, for ALL, Andean Latin America had the fastest increase in ASDR from 1990 to 2017 (EAPC=1.59, 95% CI 1.46 to 1.72) [10].

Another relevant aspect is the small number of hematologists nationwide. According to what was presented in the 2018 Yearbook of Health Activities and Resources, 130 hematologists where registered in Ecuador for that year. In the 24 provinces, 11 did not have this specialist, and 84.62% were centralized in Pichincha (30%), Guayas (32.31%), and Manabí (22.31%). This information may explain why the Amazon region has the lowest number of deaths reported, along with the lack of availability to relevant tools such as cytogenetics, molecular biology, immunohistochemistry, PET-TC, among others. In this context, health authorities must take action to overcome the reduced access to hematology services in the majority of the provinces. Besides, there is reduced availability of bone marrow transplant (BMT) centers in Ecuador, as only one institution in Guayaquil offers this service [11]. This last aspect is preoccupying, especially when many deceased patients have HN in which BMT is the first line of treatment. Additionally, the lack of access to hematology in provinces with higher poverty rates concerns us as poverty by itself is related to increased mortality in cancer in both adults and pediatric patients [12, 13]. Similarly, some ethnic groups can be affected by this unequal distribution of access, such as indigenous communities in Coastal, Andean and Amazonic regions and the afro-descendant population located north of the Coastal region. In line with this suggestion, the association between ethnicity and poverty could also negatively impact the survival of children with acute lymphoblastic leukemia and other types of adult cancers [14, 15].

## Conclusion

In Ecuador, during 2019, approximately eight people died due to HN by 100000 inhabitants, which increases to 43 defunctions by 100000 inhabitants in the population aged 60 years or more. The neoplasms with the highest number of patients deceased corresponds to Non-Hodgkin lymphomas, similar to other reports globally.

These results should be analyzed considering some deficiencies in the Ecuadorian health system as well as in the national registry, such as the reduced number of hematologists and the insufficient access to more specific diagnostic tools. Therefore, we suggest conducting more studies to understand better, how HN affects the Ecuadorian population.

## Figures and Tables

**Table 1.** Number of patients classified according to hematological neoplasm

ICD-10	Hematological neoplasm	Males		Females		Total		Age		YLL males	YLL females
		n	%	n	%	n	%	m	IQR		
**C81**	Hodgkin lymphoma	42	64.62	23	35.38	65	100	61	41	937	461.30
**C82**	Follicular lymphoma	9	52.94	8	47.05	17	100	70	24.5	1.95	176.80
**C83**	Non-Follicular lymphoma	60	50.00	60	50	120	100	68	22.5	827	1023
**C830**	Small cell B-cell lymphoma	4	66.67	2	33.33	6	100	69.5	38.25	55.4	21.4
**C831**	Mantle cell lymphoma	4	100.0	0		4	100	69.5	25	26.4	
**C833**	Diffuse large B-cell lymphoma	30	50.85	29	49.15	59	100	68	24	322	389.8
**C835**	Lymphoblastic lymphoma	7	38.89	11	61.11	18	100	58.5	57	217.2	357.2
**C837**	Burkitt lymphoma	2	66.67	1	33.33	3	100	42	42	39.2	38.2
**C839**	Other non-follicular lymphoma	13	43.33	17	56.66	30	100	69.5	17.25	166.8	216.4
**C84**	Mature T/NK-cell lymphomas	19	67.86	9	32.14	28	100	53	32.5	414.50	201.90
**C85**	Other and unspecified types of non-Hodgkin lymphoma	189	53.69	163	46.30	352	100	68	22	2014.50	2290.30
**C86**	Other specified types of T/NK-cell lymphoma	3	75.00	1	25	4	100	45.5	23	58.50	45.10
**C88**	Malignant immunoproliferative diseases	2	100.0	0	0	2	100	NC	NC	0	NC
**C90**	Multiple myeloma and malignant plasma cell neoplasms	100	52.08	92	47.91	192	100	67	19.5	694	1160.2
**C900**	Multiple myeloma	98	53.26	86	46.73	184	100	67	19.75	631.8	1093.2
**C901**	Plasma cell leukaemia	0	0.00	3	100	3	100	62	27	0	53.6
**C902**	Extramedullary plasmacytoma	2	66.67	1	33.33	3	100	51	51	62.2	0
**C903**	Solitary plasmacytoma	0	0.00	2	100	2	100	NC	NC	0	13.4
**C91**	Lymphoid leukaemia	174	52.73	156	47.27	330	100	37.5	49	6344.8	5886.2
**C910**	Acute lymphoblastic leukaemia	138	49.82	139	50.18	277	100	27	44	5843.8	5514.8
**C911**	Chronic lymphocytic leukaemia of B-cell type	16	64.00	9	36	25	100	76	17	0	152.8
**C913**	Prolymphocytic leukaemia of B-cell type	1	50.00	1	50	2	100	NC	NC	14.6	63.2
**C915**	Adult T-cell lymphoma/leukaemia	1	100.0		0	1	100	NC	NC	67.6	0
**C919**	Lymphoid leukaemia, unspecified	18	72.00	7	28.00	25	100			418.8	155.4
**C92**	Myeloid leukaemia	139	55.38	112	44.62	251	100	58	36	2614.2	3221.4
**C920**	Acute myeloblastic leukaemia	75	53.57	65	46.43	140	100	57.5	34	1690	1821
**C921**	Chronic myeloid leukaemia, BCR/ABL-positive	30	66.67	15	33.33	45	100	70	30	388	275
**C922**	Atypical chronic myeloid leukaemia, BCR/ABL- negative	1	100.0		0.00	1	100	NC	NC	0	0
**C923**	Myeloid sarcoma	1	25.00	3	75.00	4	100	33.5	36	59.6	115.6
**C924**	Acute promyelocytic leukemia	3	25.00	9	75.00	12	100	37.5	27	110.8	387.8
**C925**	Acute myelomonocytic leukaemia	0	0.00	2	100.0	2	100	NC	NC	0	22.4
**C927**	Other myeloid leukaemia	1	100.00	0	0.00	1	100	NC	NC	0	0
**C929**	Myeloid leukaemia, unspecified	28	60.87	18	39.13	46	100	65	37.75	365.8	599.6
**C93**	Monocytic leukaemia	5	71.43	2	28.57	7	100	79	25	11.5	4.20
**C946**	Myelodysplastic and myeloproliferative disease, not elsewhere classified	2	66.67	1	33.33	3	100	NC	NC	43	12.10
**C95**	Leukaemia of unspecified cell type	40	48.78	42	51.22	82	100	67	41	516	985.20
**C96**	Other and unspecified malignant neoplasms of lymphoid, haematopoietic and related tissue	3	33.33	6	66.67	9	100	62	39	79.5	93.60
**Total**		787	53.83	675	46.17	1462	100	62	34	19761	12307.8

ICD-10, International Classification of Diseases; m, median, IQR, interquartile range; *

**Table 2.** Crude mortality rate and age standardized rate of hematological neoplasm

	All age population		Population aged ≥ 60 years		Population aged 40 to 59 years		Population aged 20 to 39 years		Population aged < 20 years		Mortality rates				
Province code	n	%	N	%	n	%	N	%	n	%	CMR*	ASMR1*	ASMR2*	ASMR3*	ASMR4*
**1**	85	5.8	55	6.9	16	4.9	6	3.6	8	4.8	9.8	55.7	9.8	2.1	2.5
**2**	23	1.6	13	1.6	9	2.8	0	0.0	1	0.6	11.0	49.8	25.0	0.0	1.1
**3**	23	1.6	13	1.6	4	1.2	2	1.2	4	2.4	8.3	40.9	8.7	2.3	3.5
**4**	18	1.2	15	1.9	2	0.6	0	0.0	1	0.6	9.7	64.9	5.1	0.0	1.5
**5**	40	2.7	20	2.5	8	2.5	9	5.4	3	1.8	8.3	38.4	9.4	6.3	1.5
**6**	37	2.5	22	2.8	4	1.2	4	2.4	7	4.2	7.1	33.7	4.2	2.6	3.4
**7**	61	4.2	35	4.4	14	4.3	6	3.6	6	3.6	8.6	45.8	9.3	2.7	2.3
**8**	37	2.5	13	1.6	7	2.2	9	5.4	8	4.8	5.8	23.5	6.4	4.9	2.8
**9**	330	22.6	155	19.5	83	25.5	40	24.0	52	31.1	7.6	34.0	8.8	3.0	3.3
**10**	60	4.1	48	6.0	1	0.3	8	4.8	3	1.8	12.8	88.6	1.1	5.6	1.7
**11**	56	3.8	35	4.4	10	3.1	5	3.0	6	3.6	10.8	51.5	10.6	3.3	3.0
**12**	56	3.8	25	3.1	14	4.3	8	4.8	9	5.4	6.1	28.6	7.8	3.0	2.4
**13**	114	7.8	61	7.7	33	10.2	9	5.4	11	6.6	7.4	36.1	10.4	2.0	1.8
**14**	10	0.7	7	0.9	0	0.0	0	0.0	3	1.8	5.2	53.1	0.0	0.0	3.2
**15**	5	0.3	4	0.5	0	0.0	1	0.6	0	0.0	3.8	42.1	0.0	2.6	0.0
**16**	8	0.5	5	0.6	1	0.3	0	0.0	2	1.2	7.2	60.9	5.4	0.0	4.0
**17**	346	23.7	199	25.0	75	23.1	47	28.1	25	15.0	10.9	56.4	10.8	4.6	2.3
**18**	55	3.8	29	3.6	21	6.5	3	1.8	2	1.2	9.4	39.9	17.1	1.6	1.0
**19**	8	0.5	5	0.6	1	0.3	1	0.6	1	0.6	6.8	54.9	5.4	2.8	1.8
**20**	2	0.1	0	0.0	2	0.6	0	0.0	0	0.0	6.2	0.0	27.2	0.0	0.0
**21**	3	0.2	2	0.3	0	0.0	1	0.6	0	0.0	1.3	12.3	0.0	1.4	0.0
**22**	7	0.5	1	0.1	3	0.9	2	1.2	1	0.6	4.4	10.5	11.4	4.3	1.3
**23**	46	3.1	21	2.6	12	3.7	2	1.2	11	6.6	10.2	50.9	13.8	1.4	6.1
**24**	32	2.2	13	1.6	5	1.5	4	2.4	10	6.0	8.2	35.9	6.7	3.4	6.1
**Total**	1462	100	796	100	325	100	167	100	174	100	8.5	43.3	9.3	3.2	2.6

**ASMR1, age standardized mortality rate by population aged 60 years or more; ASMR2, age standardized mortality rate by population aged 40 to 59 years; ASMR3, age standardized mortality rate by population aged 20 to 39 years; ASMR4, age standardized mortality rate by population aged < 20 years; CMR, crude mortality rate;**

*, by 100000 inhabitants.
